# Life Satisfaction among Unaccompanied Refugee Minors: Associations with Traumatic Events and Daily Hassles

**DOI:** 10.1007/s40653-023-00579-1

**Published:** 2024-01-03

**Authors:** Anne Kristine Solhaug, Brit Oppedal, Espen Røysamb, Rachel Calam

**Affiliations:** 1 Department of Childhood and Families, Norwegian Institute of Public Health, Oslo, Norway; 2 Promenta Research Center, Department of Psychology, University of Oslo, Oslo, Norway; 3 Division of Psychology and Mental Health, University of Manchester, Manchester, UK

**Keywords:** Unaccompanied refugee minors, Life satisfaction, Daily hassles, Traumatic events, Asylum status

## Abstract

While there is a growing literature about mental health problems among unaccompanied asylum-seeking and refugee minors (URMs), far less is known about their wellbeing. Such information is important as a subjective sense of wellbeing is associated with a variety of positive health and psychosocial outcomes. The aim of this study was to examine life satisfaction and the association with traumatic events, daily hassles, and asylum status among URMs in Norway. We collected self-report questionnaire data from URMs living in Norway (*n* = 173, 90.80% male, 71.1% from Afghanistan). Mean age was 16.62 (*SD* = 1.74) years, and they reported clinically relevant post-traumatic stress symptoms. We explored variation in life satisfaction, URM-specific daily hassles and traumatic events. We also investigated a mediation model, in which we assumed that daily hassles mediated the association between traumatic events and life satisfaction. The participants reported low life satisfaction (*M* = 4.28, *SD* = 2.90, 0–10 scale). A negative outcome of the asylum process and URM-specific daily hassles were associated with reduced life satisfaction. URM-specific daily hassles accounted for the relation between traumatic events and life satisfaction. The youth had been exposed to several traumatic events yet the effect of these on life satisfaction appeared indirect, via an increase in URM-specific daily hassles. Reducing the number of, or help URM cope with, URM-specific daily hassles may increase their life satisfaction.

## Introduction

Unaccompanied asylum-seeking minors are children and youth below the age of 18 seeking protection in a foreign country, without being accompanied by a legal adult caretaker (The Norwegian Directorate of Immigration, n.d). If their asylum application is granted, they are referred to as refugees. The children and youth face a series of adversities, such as exposure to accumulated traumatic events before and during flight, an unfamiliar asylum process, and acculturation-related stressors after resettlement, which increase the risk for mental health problems (Jensen et al., 2015; Keles et al., 2016a; Vervliet et al., 2014). In the years following resettlement, many do well in spite of all these hazards (Keles et al., 2016b), but a substantial proportion of the children and youth continue to suffer from high levels of mental distress several years after resettlement (Jensen et al., 2019; Vervliet et al., 2014).

To understand mental health status in the population, we need knowledge and assessment about both illbeing and wellbeing (Iasiello & Van Agteren, 2020). While we know that mental distress is negatively associated with aspects of wellbeing (Fergusson et al., 2015; van der Boor et al., 2020), less is known about the combined impact of war-related traumatic exposure and asylum-seeking and refugee context-specific daily hassles on wellbeing outcomes among unaccompanied asylum-seeking and refugee minors (URMs). Promoting wellbeing is protective for mental health problems (Fergusson et al., 2015), and is valuable both for the individual’s functioning and for society (Diener et al., 2018). Among URMs exposed to past and ongoing hardships, increased wellbeing can enable resilience and protect from concurrent and future stress (Oppedal et al., 2022). The overall aim of this study is to generate knowledge about the associations between wellbeing, pre-migration traumatic events, and URM-specific daily hassles in a sample of asylum-seeking and resettled URMs. As all of the participants in the present study share the same history of coming to Norway as minors, we refer to them as minors, even if a few of them have reached the age of majority.

## Life Satisfaction

There are several related approaches to understand and measure wellbeing. *Quality of life* is a broad-ranged construct frequently used in the literature, and includes social, economic and subjective indicators of wellbeing (Diener & Suh, 1997). *Subjective wellbeing* is a subjective, overall evaluation of life, encompassing positive and negative affect, and life satisfaction (Diener & Suh, 1997). In the current work, we refer to *life satisfaction* to understand a person’s appraisal of life as a whole, tapping on the cognitive aspect of subjective wellbeing (Diener et al., 2018).

A systematic review of adult asylum seekers and refugees concluded that strong social networks and social integration promoted quality of life (van der Boor et al., 2020), whereas cross-sectional studies have identified aspects of the asylum- and refugee situation associated with lower quality of life. These include exposure to traumatic events and multiple losses of family members (Hengst et al., 2018), fleeing home country because of war and persecution (Myhrvold & Småstuen, 2019), and a long asylum process (Laban et al., 2008). In general, asylum seekers and refugees report low quality of life (Gottvall et al., 2019; Leiler et al., 2019; Mölsä et al., 2014).

Research on the wellbeing of the asylum-seeking and refugee population is just in the beginning. Hence there is a lack of knowledge about risk and protective factors among asylum-seeking and refugee children and youth in general, and URMs in particular. Wellbeing is not frequently studied in the literature about URMs and more fragmented studied among children with migrant background (Bajo Marcos et al., 2021). One study of resettled URMs showed high levels of life satisfaction, which was considered a potential resilience factor (Oppedal et al., 2022). Seglem et al. (2014) compared resettled URMs with adolescents with Norwegian-born and foreign-born parents. They found equal levels of life satisfaction, even though URMs reported more daily hassles and depressive symptoms than the other two groups. When daily hassles and coping strategies were controlled for in the analyses, URMs were more satisfied with life. In contrast, a study from Austria found that URMs reported lower life satisfaction in most domains, but significantly higher life satisfaction in relation to school, compared to an adolescent norm population (Huemer et al., 2011). Hence, there are inconsistent results regarding the level of life satisfaction among URMs.

## Pre-Migration Traumatic Events and Stress Reactions

A traumatic event involves “exposure to actual or threatened death, serious injury or sexual violence” (American Psychiatric Association, 2013). These events are often followed by physiological and psychological responses to the threat, such as the adaptive flight, fight and freeze responses. For some, the reactions can manifest as symptoms of post-traumatic stress disorder (PTSD), such as intrusive symptoms, avoidance behavior, hyperarousal, and alteration of mood and cognition. Exposure to traumatic events years ago can be associated with on-going increased reactivity of the autonomic system (McEwen, 2000). A developing brain is more sensitive to such experiences than a fully matured adult brain, and this can give rise to severe mental health problems (Perry et al., 1995). Several studies confirm that childhood adversities influence adolescents’ health and wellbeing (Flaherty et al., 2013; Mc Elroy & Hevey, 2014), and that cumulative risk factors predict more adverse health consequences than single risk factors (Evans et al., 2013; Flaherty et al., 2013; Kubiak, 2005; Montgomery, 2010). Exposure to traumatic events and accordingly post-traumatic stress symptoms are risk factors for lower quality of life, both among refugees and people living in war-affected communities (Matanov et al., 2013). Studies of asylum seekers and refugees have found that the association between trauma exposure and quality of life was mediated by mental distress (Araya et al., 2007; Hengst et al., 2018) and post-migration stressors (Dangmann et al., 2021). In the present study, we expand on these findings by examining the impact of URM-specific daily hassles on the association between traumatic events and life satisfaction.

## Daily Hassles and Asylum Stress

In addition to war- and disaster-related traumatic experiences and other major negative life events, URMs face a host of daily hassles (Jensen et al., 2019; Keles et al., 2016a). In the 1980’s, scholars in the field of stress and coping emphasized the role of proximal daily hassles as predictors of psychological problems, in addition to major negative life events. In their seminal work, Kanner et al. (1981) distinguished between major life events and daily hassles, which they defined as “the irritating, frustrating, distressing demands that to some degree characterize everyday transactions with the environment” ( p. 3).

Keles et al. (2016a) suggested to separate between *general daily hassles* and *acculturation-specific daily hassles* when examining daily hassles among URMs. General hassles are experienced by everyone, such as conflicts with social network members and school-related problems. Acculturation-specific hassles are specifically related to having an immigrant or refugee status. Such hassles can be culture-related conflicts with ingroup members, such as making decisions about own ethnic identity, or frustration related to membership in an outgroup, such as not understanding their behavior or jokes (Keles et al., 2016a; Lay & Nguyen, 1998). The hassles can also be associated with the acculturation process, such as frustrations due to not understanding the new language.

Among URMs, both domains of hassles (general and acculturation-specific) had unique associations with depressive symptoms, both cross-sectionally and longitudinally, beyond the effect of pre-migration traumatic events (Keles et al., 2016a). These findings suggest that daily hassles are context specific. Asylum seekers may face daily hassles during the time they stay in reception centers that differ from hassles experienced by youth who have a residence permit. Therefore, in the present study, we apply URM-specific daily hassles to indicate hassles that are distinct to young asylum-seeking and refugee context. Presumably, the frequency and accumulation of URM-specific daily hassles have the same negative mental health outcomes as other kinds of daily hassles.

Kanner et al. (1981) demonstrated that daily hassles predicted psychological symptoms better than negative life events, shifting from focusing mainly on traumatic events as predictors of mental health problems, to more comprehensive models, which also included ongoing risk and protective factors. Miller and Rasmussen (2010) proposed a daily stressor model that integrates the direct effect of traumatic events on mental health problems and the indirect effect of traumatic events through daily hassles in conflict and post-conflict environments. Several studies have applied the same framework, and showed that different types of daily hassles accounted for the relation between traumatic events and mental health (Hou et al., 2019). This shows that among refugees and asylum-seekers, daily stressors in the context of the receiving country can have a stronger impact on mental health than their pre-migration traumatic experiences (Hou et al., 2019). However, in contrast to studies on the association between trauma, daily stressors, and symptoms of PTSD, depression, and anxiety, the daily stressors did not mediate the association between trauma and wellbeing. Hou et al. (2019) identified few studies on this matter, and therefore recommended to investigate associations between daily stressors and wellbeing in other populations.

Different contexts reflect different hassles. In a high-income country such as Norway, the welfare state ensures that URMs’ basic needs are met, yet the new life as an asylum-seeker or refugee poses many challenges and stressors. This differs from the context of conflict and post-conflict where daily hassles may be more related to material stressors, such as ensuring food, water, and shelter (Miller & Rasmussen, 2010). A meta-analysis of daily stressors and mental health among forced migrants showed that type of host country (developing or developed) moderated the effect size between everyday life experiences and mental health, with the strongest effect sizes for developing host countries (Hou et al., 2019). This implies that the hassles in developing countries are more severe to the individual’s mental health. In the present study, we expand on the daily stressor model with life satisfaction outcomes. As demonstrated in several studies, traumatic events and daily hassles reduce different aspects of wellbeing (Dangmann et al., 2021; Matanov et al., 2013; Oppedal et al., 2022; Seglem et al., 2014), but we do not know how these associations manifest within the daily stressor framework in the context of URMs resettling or seeking asylum in a high-income country.

A devastating stressor in the post-migration environment is the asylum application process itself, causing high levels of distress among URMs and other asylum seekers (Heptinstall et al., 2004; Jakobsen et al., 2017). The asylum process is beyond the individual’s control and with major consequences for the future. Oppedal et al. (2020) found that it was not the length of the asylum process that predicted mental health problems among resettled URMs. They suggested that it was the accumulation of asylum-related stressors, and the uncertainty of the asylum application that were associated with depression.

Most of the participants in the present study came to Norway during 2015–2016, referred to as the European migrant crisis (Spindler, 2015). Following the high numbers of asylum-seekers and refugees, including URMs, governments in many European countries implemented harsh immigration policies. These especially affected URMs from Afghanistan, which was one of the largest national groups. Among other new measures, many of them were returned to “internal protection” implying that a minor asylum-seeker from e.g. Kandahar, could risk being returned to e.g. Kabul for protection, even if they had no known network or support there (Schultz, 2018). We therefore included various outcomes of the participants’ asylum status and asylum-specific daily hassles when examining URM’s life satisfaction.

## The Present Study

The overall aim of this study was to get knowledge about how the asylum-seeking and refugee context of URMs in Norway may impact URMs' sense of life satisfaction. In Norway, support for URMs is highly regulated and provided by public institutions, such as the Child Welfare Service. This implies a higher degree of adult supervision and support than in URM contexts in other European countries.

We need new information about life satisfaction in a group of children and youth in a particularly vulnerable situation. Such information may inform clinicians and policy makers and provide new knowledge about context specific predictors of life satisfaction. While traumatic exposure and daily hassles are well-known and consistent predictors of depression and PTSD among URMs (Jensen et al., 2019; Vervliet et al., 2014), less is known about their association with life satisfaction.

In the present study, two groups of refugee advisors have been involved. A group of URMs advised us regarding the implementation of the Coping among Asylum-Seeking and Refugee Minors (CaSARM)-study and contributed to the development and accommodation of the measures of URM-specific traumatic events and daily hassles. This group of refugee advisors is referred to as RA-A. Another group of young, adult refugee advisors (RA-B) helped us to better understand the current study topic. Both advisor groups were organized as focus groups, and the advisors received a gift voucher for their contribution. The advisors contributed to deepen our understanding of their experiences with flight, seeking asylum and resettling in Norway without the support of parents. We were strongly advised by RA-B that asylum status should be included in the study questions in order to understand variation in the associations between traumatic events, daily hassles and life satisfaction. We included three possible aspects of asylum status: residence permit, waiting for a decision and still hoping for a positive outcome of the asylum application, and rejection. Based on the experiences of the refugee advisors and the literature review, we hypothesized that:

- There is variation in the level of life satisfaction and URM-specific daily hassles among URMs with different asylum status. URMs with rejection have lower life satisfaction and report more daily hassles than URMs with a pending asylum claim or with residence permit.
- The accumulation of pre-migration traumatic events, URM-specific daily hassles and asylum status predict lower life satisfaction.
- URM-specific daily hassles mediate the association between accumulated pre-migration traumatic events and life satisfaction.

## Method

### Procedure

The data for the present study was based on data from the first wave of the CaSARM-study, which was an intervention study, aiming to implement and evaluate a low-threshold mental health program, Teaching Recovery Techniques (TRT) (Yule et al., 2013). The Regional Committees for Medical and Health Research Ethics approved the study. Participation was contingent on written consent. When the participant was younger than 16 years, their assigned representative or legal guardian gave the consent, in addition to the child’s verbal consent. The participants were informed that participation in the study was voluntary, and we emphasized that it would not impact their asylum application.

Children and youth (> 10 years) speaking Pashto, Dari, Arabic, Somali or Tigrinya were the target group for the study. The majority of URMs in Norway at that time spoke these languages, thus we offered the group-based TRT-intervention in the above-mentioned languages. The participants were recruited from three types of care conditions, i.e. care centers for asylum-seeking URMs younger than 15 years, reception centers for URMs 15 years and older, and residence municipalities for URMs with residence permit. The centers and municipalities were located from the northernmost to the southernmost parts of Norway. The research team visited all the sites and met the youth, caretakers and staff, in a group meeting with interpreters present, to inform them about the study. The youth who agreed to participate in the study and the TRT-course were asked to complete a checklist of PTSD-symptoms, *Children’s Revised Impact of Event Scale (CRIES- 8) *(Smith et al., 2003). We invited youth with a symptom-level above the clinical cutoff score (≥ 17), because the TRT-intervention was recommended for children and youth enduring high levels of distress. The staff was informed about their distress and their initiative to attend the TRT-program.

**Fig. 1:**
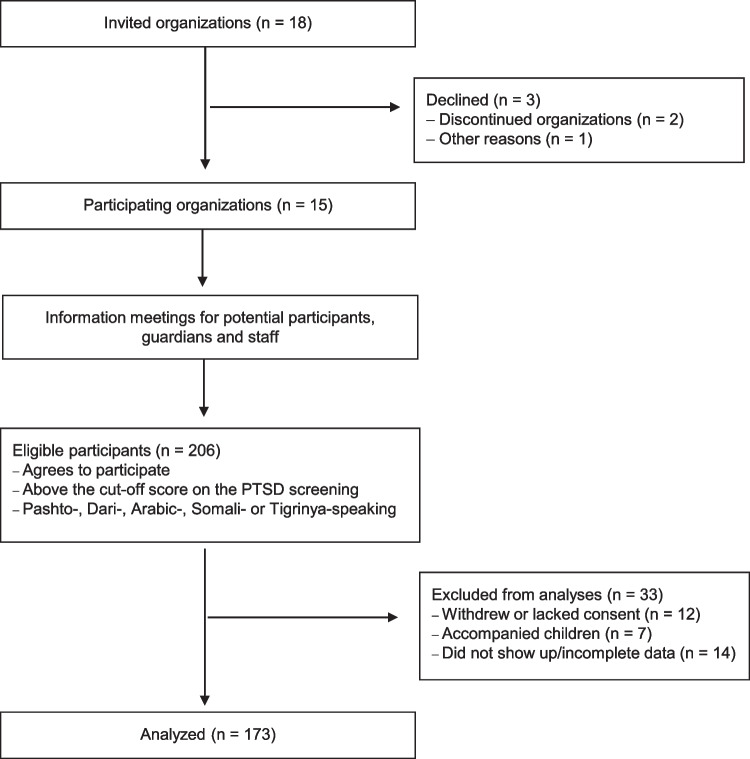
Recruitment and procedure

Altogether, 206 youth were interested and registered for participation. Out of this, 84% (*n* = 173) were present at the first data collection and included in the present study. See Fig. 1. The participants met in groups in places familiar to them in their local communities to fill in the self-report questionnaire (pen and paper). The research team was present at the data collection. Trained bilingual research assistants who read, explained and helped the participants in their mother tongue, assisted the participants.

### Participants

The average age of participants in this study was 16.62 years (*SD* 1.74, range 11 – 23), and they had mostly been in Norway between 1–2 years (49.7%). Most of the participants were males (90.8%) and originated from Afghanistan (71.1%, *n* = 118). Half of the sample lived in facilities for asylum-seeking children (50.3%), either in care centers (15.6%) or in URM-reception centers (34.7%). The remaining participants were resettled in municipalities.

While all the participants had lodged their asylum-application, they were in different stages of its processing. Some were waiting, either for the asylum interview or for a decision (18.4%), some had their application rejected (19.0%) and others had received residency permits (62.6%). They were either resettled in a municipality or waiting at the reception center to be resettled. Three persons, however, were resettled, but their asylum claim had been rejected. This is because the Norwegian Government cannot return children (< 18 years) to their home country without a caregiver. See Table 1.

**Table 1:** Sample Characteristics

	Total sample		Asylum seekers		Refugees	
	*n*	%	*n*	%	*n*	%
Gender						
Male	157	90.8	87	100	70	81.4
Female	16	9.2	-	-	16	18.6
Country of origin						
Afghanistan	118	71.1	79	96.3	39	46.4
Eritrea	24	14.5	-	-	24	28.6
Syria	11	6.6	-	-	11	13.1
Other	13	7.8	3	3.7	10	11.9
Care condition						
Reception center	60	34.7	60	69.0	-	-
Care center	27	15.6	27	31.0	-	-
Municipality	86	49.7	-	-	86	100
Asylum status						
Rejection	28	19.0	25	34.7	3	4.0
Waiting	27	18.4	27	37.5	-	-
Residence	92	62.6	20	27.8	72	96.0

*N* = 173. Mean age asylum seekers 15.76 (*SD* 1.54), mean age refugees 17.53 (*SD* 1.47)

### Measures

Professional translators provided translations of the questionnaires in all five study languages (Pashto, Dari, Arabic, Somali and Tigrinya). Our bilingual research assistants revised these translations in collaboration with the research team, to assure the correct nuances of words and expressions. The refugee advisors (RA-A) piloted the measures.

*Life satisfaction* was measured by an adapted version of the Cantril ladder (Cantril, 1965) visualized with ten steps, 1–10. The participants were instructed to visualize the ladder as a picture of their life right now. The top of the ladder represented the best possible life, and the bottom step the worst possible life. The participants checked the step that best reflected how they felt about life right now. For the purpose of comparability with other studies, we transformed the scale to 0–10, (raw scale score-1) / 9*10. The measure has shown good reliability and convergent validity among adolescents (Levin & Currie, 2014). A score of ≥ 6 distinguished high from low life satisfaction (Inchley et al., 2016).

*The Traumatic Events Checklist* was based on The Stressful Life Events measure (Bean et al., 2004), but revised and accommodated in close collaboration with our refugee advisors, RA-A. In this way, the events were relevant for asylum seekers and refugees coming to Norway at that time. The participants checked whether they had been (1) or had not been (0) exposed to 21 war- and disaster related traumatic events, such as “have you experienced that someone you cared for was killed in war or during flight?”, “have you fled in a boat over sea?” and “have you experienced that other hit or kicked you, shot at you or in other ways tried to harm you physically?”. Total score ranged from 0 – 21.

*The Daily Hassles Measure* was developed for the CaSARM-study, revised and accommodated to the refugee advisors' (RA-A)experience. The scale consisted of 22 context-specific items, reflecting URM-specific hassles, that is hassles generated by the asylum-seeking or resettlement context. Participants living in reception centers for asylum seekers answered nine questions mainly related to their asylm-seeking situation, such as “I get stressed when others get their asylum application rejected”, “I worry if I will get a residence permit”, and “I am afraid of being deported by the police”. Youth living in the municipalities answered nine items targeting hassles relevant for the refugee- and resettlement context, such as “Norwegians look down on people from my home country”, “heavy workload at school”, and “difficulties making Norwegian friends.” Four items were similar for both contexts such as: “No one can help me with my problems”, “troubles or conflicts with the adults where I live”, “troubles or conflicts with friends”, and “worries about my family abroad.” The participants reported whether they had never (1), sometimes (2), often (3) or almost all the time (4) experienced each hassle. We standardized each scale with the different hassles and merged the two scales into one.

*Asylum status* was based on the participants’ self-reported asylum status, grouped into 1) rejection, either temporary leave to remain or permanent rejection 2) waiting for interview, application or appeal, 3) residence. We dummy-coded this into rejection (0) and waiting/residence permit (1).

We included age and length of stay as covariates. Age was indicated in terms of years, from 11–23. Length of stay was measured in terms of one-year units, i.e., 0 = less than a year, 1 = 1–2 years, 2 = 2–3 years and so on. Gender was not included as a covariate, because of the few participating girls.

### Statistical Analyses

Analyses were conducted with the R statistical software version 3.6.2, the Lavaan package 0.6–5 (Rosseel, 2012), and IBM SPSS version 26. The fit indices of the mediation model were examined by the Root Mean Square Error of Approximation (RMSEA), Tucker- Lewis Index (TLI), Comparative Fit Index (CFI) and the Chi-Square (*X*^*2*^). RMSEA ≤ 0.08 indicates an adequate fit, and ≤ 0.05 a close fit (Browne & Cudeck, 1993). TLI and CFI should be > 0.95 (Hu et al., 1999). Age and length of stay weres only correlated with daily hassles, thus we controlled for age and length of stay on the mediator in mediation analysis.

Missing data ranged from 0 – 25%, mainly associated with the Cantril ladder (25%). There were more missing data among URMs living at reception centers compared to the resettled URMs. We applied mean sum scores for the daily hassles measure and the trauma checklist. We estimated the mediation model applying Maximum Likelihood Estimation (ML) and applied bootstrapping method with 1000 iterations to provide bias corrected standard errors in all analyses.

## Results

### Descriptive Statistics

Table 2 shows descriptive statistics for, and correlations between, the study-variables for the overall sample. Life satisfaction correlated negatively with traumatic events and daily hassles but was not related to length of stay and age. The average life satisfaction score on the Cantril ladder was 4.28 (*SD* 2.90). As much as 76.7% considered their life satisfaction as low (Cantril score < 6, not in the Table). Furthermore, the youth had been exposed to an average of 11.38 (*SD* 4.47) war- and disaster-related traumatic events, such as “someone in your family was severely injured during war” (57.2%) and “experienced armed combat (such as shooting, airstrikes, bomb explosions etc.) at first hand” (67.6%).

**Table 2:** Descriptive Statistics and Correlations

	*n*	*M*	*SD*	1	2	3	4	5	6
1. Life Sat	129	4.28	2.90	-					
2. Trauma	173	11.38	4.47	-0.23**	-				
3. Hassles	149	0.00	1.00	-0.41**	0.28**	-			
4. Asylum	147	0.81	0.39	0.40**	-0.16	-0.31**	-		
5. Stay	149	1.27	0.87	-0.02	-0.05	0.17*	0.11	-	
6. Age	165	16.62	1.74	-0.16	-0.09	0.25**	-0.02	0.66**	-

*Life Sat *life satisfaction, *Trauma *traumatic events, *Hassles *standardized measure of daily hassles, *Asylum *asylum status (dummy coded, 0 = rejection, 1 = waiting/stay)

*p < 0.05; **p < 0.01

The youth were struggling with several ongoing hassles. A large majority (80.4%) reported that they “frequently” or “almost always” worried about their family abroad. Asylum-seeking URMs reported most frequently that they became stressed “when other asylum seekers had their asylum application rejected” (80.0%), and that they ruminated about “getting residence permit in Norway” (75.7%). Resettled URMs reported that school-related problems were most frequent. More than half of them, 53.5%, reported that they “often” or “most of the time” had experienced “heavy pressure to do well in school.”

### Group Mean Differences by Asylum Status

Table 3 shows the means and standard deviations of the main study-variables for each asylum status group, and the result of the one-way Anova significant tests. URMs in the rejected group differed significantly from both those in the waiting and the residence groups in life satisfaction and daily hassles. There were no significant differences between the waiting group and the residence group in life satisfaction and daily hassles-scores. The number of traumatic events reported by the youth was independent of their asylum status. Based on these findings, we decided to dummy code asylum status, distinguishing between URMs with rejection (0) and URMs who were waiting and with residence permit (1).

**Table 3:** Means, Standard Deviations and One-Way ANOVA

	Rejection		Waiting		Residence		ANOVA	
	*M*	*SD*	*M*	*SD*	*M*	*SD*	*F- ratio*	*df*
Life Sat	1.70	2.14	4.44	3.16	5.00	2.65	11.67**	2,118
Hassles	0.65	0.79	-0.25	0.96	-0.11	1.04	7.41**	2,137
Trauma	12.96	4.66	12.04	3.87	11.00	4.27	2.46	2,144

*N* = 121, *ANOVA* analysis of variance, *Life Sat* life satisfaction, *Hassles *standardized measure of daily hassles, *Trauma *traumatic events

**p < 0.01

Rejected URMs reported on average 2.75 Cantril ladder scores (life satisfaction) lower than URMs who were waiting for the result of the asylum claim, and 3.30 scores lower than URMs with a residency permit. The standardized mean of daily hassles was 0.90 SD higher among rejected URMs compared to URMs who were waiting, and 0.76 SD higher among URMs with residence permit compared to rejected URMs.

### Predictors of Life Satisfaction

Table 4 shows that asylum status and daily hassles were the only significant predictors of life satisfaction. Traumatic events did not predict life satisfaction in this model.

**Table 4:** Regression Analysis

	*B*	95% CI for B		*SE B*	β	R^2^
		LL	UL			
						0.31
Constant	7.29*	1.21	12.44	2.90		
Trauma	-0.10	-0.22	0.03	0.06	-0.15	
Daily Hassles	-0.83*	-1.35	-0.28	0.27	-0.28*	
Asylum Status	2.31**	1.16	3.33	0.55	0.31**	
Age	-0.26	-0.57	0.10	0.17	-0.15	
Length of Stay	0.29	-0.42	1.03	0.37	0.09	

Confidence intervals and standard errors based on 1000 bootstrap samples

* CI* confidence interval, *LL *lower limit, *UL *upper limit, *SE *St. Error

*p < 0.05; **p < *0.01*

### The Indirect Effect of Traumatic Events on Life Satisfaction through Daily Hassles

**Fig. 2:**
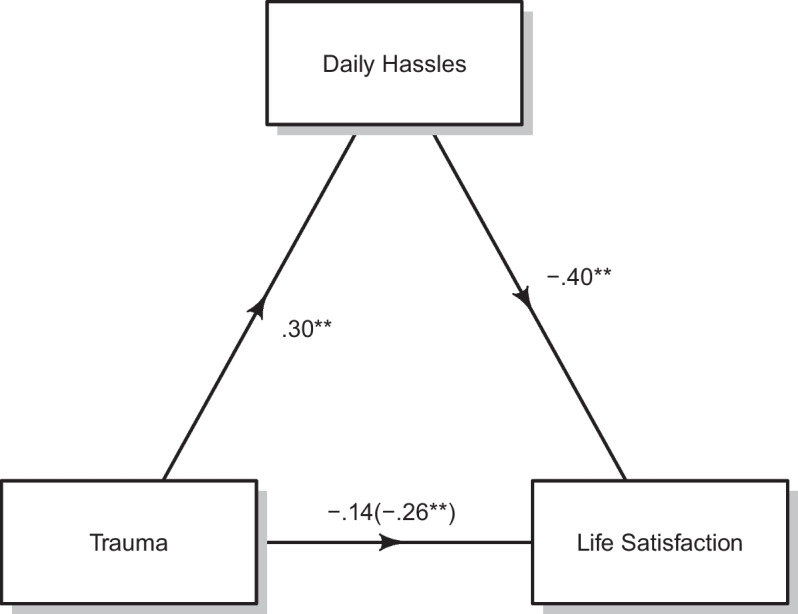
The Indirect Effect of Trauma on Life Satisfaction through Daily Hassles. *Note*: Standardized Regression Coefficients. Total effect in parenthesis. We controlled for age and length of stay in relation to daily hassles. Age was significantly associated with daily hassles β = .28, *p* =.01. Length of stay was not. **p < .01

The estimated mediation model yielded good fit indices, *X*^*2*^ (2, 173) = 2.82, *p* = 0.24, RMSEA = 0.05, 90% CI [0.00, 0.18], CFI = 0.98, and TLI = 0.93. Figure 2 shows that the total effect of traumatic events on life satisfaction was significant *(β* = -0.26, 95%BCI [-0.29, -0.04], *p* < 0.01). The Figure also illustrates that the direct effect of traumatic events when we included daily hassles in the model was β = -0.14, 95% BCI [-0.21, 0.03], *p* = 0.13, and no longer significant. The results of the mediation analysis showed that the effect of daily hassles on life satisfaction, (*β* = *-*0.40, 95% BCI [-0.61, -1.73], *p* < 0.01), included an indirect effect of traumatic events of *β* = -0.12, 95% BCI [-0.14, -0.03], *p* < 0.01.

## Discussion

This is the first study to investigate the associations between pre-migration traumatic events, daily hassles, and asylum status with overall life satisfaction among unaccompanied minor asylum seekers and refugees. The sample is unique and consists of URMs in all stages of the asylum process, living in different care conditions, with high levels of PTSD-symptoms, and with a variety of national and cultural backgrounds. We developed and accommodated the daily hassles-measure and the traumatic event checklist together with our refugee advisors, to make sure they were relevant for URMs coming to Norway at that time.

The findings show that URMs report low life satisfaction, many hassles in their everyday life, and exposure to a high number of traumatic events. Our findings suggest that the low life satisfaction is not solely based on their pre-migration traumatic events, but by large based on their concurrent daily hassles and asylum process. Traumatic events are associated with life satisfaction indirectly through daily hassles, showing that the existing framework of the Daily Stressor Model is relevant for URMs in high-income countries and with wellbeing outcomes. We learn that traumatic events and life satisfaction is interrelated through other mechanisms than the direct effect between traumatic events and mental health, supporting the notion of assessing both wellbeing and illbeing to understand mental health.

### Life Satisfaction

A general measure of life satisfaction can provide a snapshot of how the youth are doing, right here and now. URMs in this study reported low life satisfaction, and youth with a rejected asylum application reported the lowest levels. In Norway, the average score of life satisfaction assessed by Cantril’s ladder is 7.49 (2.54) (Helliwell et al., 2020). This is more than one standard deviation higher than reported by the youth in the present study. Although most participants in the study had clinical range PTSD-symptoms, this large difference in perceived life satisfaction compared to other Norwegians is worrisome. In contrast, Oppedal et al. (2022), found relatively high life satisfaction among resettled URMs with longer stay time in Norway. Among URMs in Austria, life satisfaction was lower than other adolescents, but this varied across different domains (Huemer et al., 2011). The researchers suggested that URMs found it easier to report problems not associated with mental health. By reporting on general life satisfaction, compared to mental health problems, they were not associated with a diagnosis, not overwhelmed by their difficulties, and not described in an unfavorable manner (Huemer et al., 2011). This imply that assessing wellbeing can provide us with new and valuable information about the youth, besides what is traditionally reported in clinical interviews, screenings and surveys.

We discussed the low life satisfaction with the refugee advisors of the present study (RA-B). They highlighted the importance of supportive friendships and family for a good life, but also how their worries and strain in everyday life negatively impacted their wellbeing. They had a good life in Norway after resettlement, although they felt that they had to cope with everything alone, both their worries in everyday life and their past dramatic experiences. Chase (2013) applied the notion of *ontological security* (Giddens, 1989) to understand and promote better wellbeing among URMs. Ontological security reflects the security that comes from predictable routines and that events have some degree of certainty. Chase (2013) showed that a coherent sense of self and future were related to wellbeing, but this was endangered by past trauma exposure, current mental health problems, the asylum system and the stigma associated with being an asylum seeker. It is likely that this can apply to the participants in the present study as well, especially for the youth with a rejected asylum claim. An inconclusive or rejected asylum application destabilizes the ontological security, threatening the need to belong, predictability, sense of self and future.

### Traumatic Events, Daily Hassles, Asylum Status, and Life Satisfaction

Traditionally, the literature has focused on pre-migration trauma exposure as the sole predictor of mental health problems among war affected populations, but a growing number of studies are now incorporating peri – and post-migration factors in their frameworks (Miller & Rasmussen, 2010). As in other studies of URMs, the participants reported that they had been exposed to many war-and disaster-related events before their arrival in Norway, and a number of daily hassles (Jensen et al., 2015). The youths’ different asylum statuses were associated with life satisfaction and perception of daily hassles, as hypothesized by our refugee advisors. Traumatic events were not associated with asylum status.

The results showing a statistically significant relation between URM-specific daily hassles and life satisfaction, are corroborated by findings from another study among resettled URMs in Norway (Seglem et al., 2014). The findings suggest a negative dose–effect relation between daily hassles and life satisfaction, meaning that the more hassles, the poorer life satisfaction. The URM-specific hassles in the present study were developed together with the refugee advisors to better reflect hassles and experiences relevant for the URMs. All hassles are taxing to the individual’s coping resources, as noted by Keles et al. (2016a), but the URM-specific hassles may be more difficult to cope with through adaptive strategies. They may be related to the situation in their home country (“worries about family abroad”), they may be caused by the asylum legislation (“my friends were deported to their home country”) or rooted in prejudice against the individual’s cultural group (“Norwegians look down on people from my home country”). Asylum status was a significant additional burden and a major source of stress, contributing to both mental health problems and reduced wellbeing, as demonstrated in other studies (Heptinstall et al., 2004; Mueller et al., 2011; Ryan et al., 2008). Both the asylum status they receive, and the URM-specific daily hassles may be perceived as beyond the individual’s control, which negatively affects life satisfaction. The URMs need therefore help and support to make sense of and exert influence of their situation.

The significant effect of traumatic events on life satisfaction mediated by daily hassles expands on the relevance of the daily hassles framework proposed for conflict and post-conflict contexts, to other contexts, such as URMs seeking asylum or resettling in a high-income country. As in Dangmann et al. (2021), this framework was relevant in a high-resource context, showing the usefulness of context-specific hassles. This implies that it is the generation and accumulation of the hassles that drive this influence, regardless of context. However, in high-income countries, such as Norway, some of these hassles are possible to overcome and reduce with support and governmental action, perhaps in contrast to the daily hassles in conflict and post-conflict contexts.

We suggest that the accumulation of traumatic events is associated with an increase in the perception of daily hassles, which again reduces life satisfaction among URMs. Whether trauma exposure puts the individual at more risk for daily hassles, tax the individual’s ability to cope with the stressors, or if they experience more subjective distress from the hassles is not possible to conclude on with cross-sectional data, but has been suggested by previous literature (Kubiak, 2005; Silove et al., 1997; Steel et al., 1999).

The underlying mechanisms are, however, connected to an accumulation of strain and stress, activating the bodily stress response. *Allostatic load* refers to the relationship between stressful environmental factors and the repeated physiological response to these stressors. This response can tear and wear on tissues and organs in the body over time (McEwen, 2000; McEwen & Stellar, 1993). The allostatic load model can inform our understanding of how the accumulation of traumatic events, daily hassles and uncertainty about asylum status, can overwhelm the physiological system and the individual’s coping capacity. The model shed light over the cost of coping with severe chronic stress over long time, mobilizing the neural, neuroendocrine, and immune system in the body on an ongoing basis (McEwen & Stellar, 1993). We did not investigate the allostatic load model in this study, but the model frames a biopsychosocial understanding of the costs of accumulation of stress, both traumatic stress and daily hassles.

### Implications

The findings from the present study show that the URMs report alarmingly low life satisfaction and face many challenges in their everyday lives. To scaffold safe developmental contexts for trauma-exposed children and youth, it is necessary with supportive caretakers practicing a trauma-informed approach (Wilson et al., 2013), and provide better access to the healthcare services (Sønsterudbråten et al., 2018).

It is, as shown in the present study, important to also identify and target daily hassles. These hassles are often nearer in time and place, can be chronic of nature, and apply to everyone regardless of trauma exposure (Miller & Rasmussen, 2010). The current study suggests that environmental stressors, such as URM-specific daily hassles, have stronger impact on life satisfaction than pre-migration traumatic events, which was also found in other studies (Dangmann et al., 2021). A focus on daily hassles, may lead us to environment – level interventions (Hou et al., 2019). The Drive to Thrive Theory (Hou et al., 2018) can be a useful framework to support the youth with their daily hassles, and increase their wellbeing. The theory emphasizes that routines and structure of everyday life determine resilience to stress, meaning that sustaining daily routines can be highly supportive. Following this line, it can be possible to adjust the URM environment, from toxic and stressing, to supportive and resilient by focusing on daily routines. URMs might have lived with their family in their home country and must establish their own structure of everyday life. In this new context, the youth’s caretakers should guide and help them to restore and adapt to everyday routines. Hopefully this can contribute to reduce some of the hassles in everyday life. Daily hassles may also have important implications for the integration process into the Norwegian society, because of the association with mental health problems and low wellbeing. However, this will not address the other stressful source; asylum status.

The asylum stress requires effort and measures at the macro level, needing a shift in policy and politics for URMs, but also for adult asylum seekers, migrants and refugees. An overall aim for the policy makers should be to ensure wellbeing for all their inhabitants (cf. the 2030 Agenda for Sustainable Development (United Nations, 2019). This includes the time processing the youths’ asylum application. We encourage additional focus on the youths’ wellbeing and mental health in this vulnerable stage of migration. It is necessary to take action to decrease the time to process asylum applications and reduce the internal protection alternative. Ideally, the whole asylum-process for URMs should be reconsidered, in accordance with the Convention on the Rights of the Child.

### Limitations

We will here address some of the limitations in the study. First, because of the cross-sectional nature of the data, our findings imply associations between the included variables rather than causal effects for which longitudinal data is needed. Longitudinal data could provide a better understanding of factors that influence URMs’ life satisfaction and how these evolve over time. Moreover, future studies should test the mediation model with longitudinal data. Second, the design of the effectiveness study, CaSARM, involved specific inclusion and exclusion criteria, constraining the number of participants. This affected the sample size and limited the power of the analyses. The sample includes URMs speaking Dari, Pashto, Arabic, Somali, and Tigrinya, and with clinical-range symptoms of PTSD. This is a representative sample of URMs coming to Norway at that time and across the language groups, yet we cannot generalize the findings beyond this. Furthermore, we only screened for PTSD-symptoms and did not establish a formal diagnosis with standard diagnostic manuals for inclusion in the CASaRM-study. This was not the aim of the study, but co-morbid anxiety/depression can confound the results. We cannot identify this and other confounding variables with the current design.

Also, there are aspects with the measurement that need to be considered. The study is based on self-reported data, which can have important biases. The participants may not wish to disclose their experiences, or they do not remember them. A prospective study of Afghan youth showed that they forgot or repressed the trauma they had been exposed to, except for witnessing military action, which was consistently recalled (Panter‐Brick et al., 2015). Panter‐Brick et al. (2015) noted that this finding may not be surprising among young people who had lived in a context with war and multiple traumas. This implies that responses to the checklist of traumatic exposure may be deflated. Errors related to self-report measures may also occur because of translated questionnaires and varying reading competence among the participants. The bilingual research assistants’ support was therefore crucial. They read, explained and helped the participants. In addition, they were acquainted with the participants’ culture and thus also functioned as cultural mediators during the data collection. However, because of the research assistants’ presence, some responses can have been biased by the social desirability effect.

Furthermore, we applied a checklist of traumatic events developed together with the refugee advisors to capture the accumulative effect of traumatic events. A checklist approach, however, does not capture if the participant experienced the same event multiple times or just one time, and we risk capturing the effect of variation in traumatic events. However, Rasmussen et al. (2018) argued that the variety and the frequency of traumatic events are conceptually and empirically correlated in war-affected populations. This implies that the actual number of traumatic events might be higher than what is shown in this study.

Finally, the Daily Hassles Scale was used to investigate the associations with both general daily hassles, asylum-related hassles and acculturation-related hassles. We investigated hassles relevant for youth living at reception centers for asylum seekers and in municipalities, developed in cooperation with the refugee advisors. This implied that the participants did not answer the same items. In this way, we targeted the most relevant hassles, took into consideration that the participants did not share the same everyday experience, and that the various hassles might have different meanings for the youth, as in the Keles et al. (2016a) study. However, the severity of the hassles might differ between the groups, and this can influence the results. To overcome this, we also conducted the analyses with the four hassles that were similar in each sub-sample. The result confirmed that also the short version of daily hassles predicted life satisfaction. We decided to keep the scale answered by both groups, to be able to capture the accumulation of daily hassles. As noted by Kanner et al. (1981), it is the accumulation of the hassles that impact the individual’s wellbeing. A comparative design with one group of trauma-exposed refugee children who have received treatment, one group who did not receive treatment and a group of non-trauma-exposed refugee children can provide a better understanding of the mechanisms behind exposure to daily hassles, and perceived life satisfaction.

## Conclusion

The aim of the study was to investigate the associations between traumatic events, URM-specific daily hassles, asylum status and life satisfaction in a sample of URMs seeking asylum and resettling in Norway. The findings from the study expand on current knowledge by highlighting the low life satisfaction in a hard to reach-population, identifying risk-factors for reduced life satisfaction, and by showing that it is the URM-specific daily hassles and the asylum process that tax their overall life satisfaction, beyond the effect of traumatic events. We explored a mediation model based on the daily stressor framework (Miller & Rasmussen, 2010) with wellbeing outcomes and in the context of post-migration. We found that even though the youth had been exposed to an accumulation of traumatic events, this was not directly associated with life satisfaction, but rather accounted for through daily hassles. The dynamic interrelation of many both past and current stressors is a heavy burden during an important developmental period, passing from childhood to adulthood. The youth do not have their caregivers available and dependent on the social welfare system for support. It is important to structure support systems and everyday routines to establish a sense of control over life and ontological security to ensure wellbeing. To address the negative effect of the asylum process on the youths’ wellbeing, national authorities are encouraged to ensure wellbeing and mental health while processing the asylum claim of unaccompanied asylum-seeking minors.

## Data Availability

The data analyzed in this study is not publicly available. Participants have not consented to data sharing.

## Abbreviations

CaASARM: Coping among Asylum-Seeking-and Refugee Minors
CRIES: Children Revised Impact of Event Scale
RA-A: Refugee Advisors A
RA-B: Refugee Advisors B
PTSD: Post-traumatic stress disorder
URMs: Unaccompanied asylum-seeking and refugee minors
